# Epigenetic reprogramming in Urothelial bladder cancer: mechanisms, biomarkers, and therapeutic opportunities

**DOI:** 10.1080/15592294.2026.2753250

**Published:** 2026-11-07

**Authors:** Qian Yuanjiang, Yang Liming

**Affiliations:** Department of Urology, Chongqing Jiulongpo People’s Hospital, Chongqing, China

**Keywords:** Urothelial bladder cancer, Epigenetic dysregulation, chromatin remodeling, Enhancer reprogramming, DNA methylation biomarkers, epigenetic therapy

## Abstract

Urothelial bladder cancer (UBC) is a molecularly heterogeneous malignancy characterized by frequent recurrence, variable progression, and treatment resistance. In addition to recurrent genetic alterations, dysregulation of chromatin remodeling, histone modifications, DNA methylation, and enhancer activity contributes to the biological diversity of UBC. This narrative review critically evaluates the role of epigenetic reprogramming in UBC and integrates mechanistic evidence with biomarker development and therapeutic investigation. Recurrent alterations involving ARID1A, KDM6A, KMT2C/KMT2D, EP300, and CREBBP can modify chromatin accessibility and transcriptional programs, although the functional consequences and strength of causal evidence vary among regulators. Preclinical studies have identified therapeutically relevant dependencies, including the relationship between KDM6A loss, PRC2-mediated repression, and EZH2 inhibition, but clinical validation of most epigenetic targets remains limited. DNA methylation represents the most extensively investigated epigenetic biomarker class in UBC. Urine-based methylation assays have shown encouraging diagnostic performance, whereas plasma cell-free DNA methylation remains an emerging approach requiring further prospective validation. Clinical studies of epigenetic therapies have produced mixed results, emphasizing the need to distinguish biological rationale from demonstrated therapeutic efficacy. Collectively, current evidence supports epigenetic alterations as important contributors to UBC biology while highlighting the need for functional validation, biomarker-selected trials, and longitudinal epigenomic studies before routine clinical implementation.

## Introduction

Urothelial bladder cancer (UBC) is one of the most common malignancies of the urinary tract and remains a major cause of cancer-related morbidity and mortality worldwide [1]. Clinically, UBC spans a heterogeneous disease spectrum ranging from non-muscle-invasive bladder cancer (NMIBC), which is characterized by frequent recurrence but often favorable short-term survival, to muscle-invasive bladder cancer (MIBC), which is associated with aggressive progression, metastatic dissemination, and poor long-term outcomes [2]. Despite advances in surgery, intravesical therapy, immunotherapy, and systemic treatment, durable disease control remains limited in a substantial proportion of patients, particularly in advanced disease [3].

Historically, the molecular pathogenesis of UBC has been explained primarily through recurrent genetic alterations affecting oncogenic signaling and tumor suppressor pathways, including mutations in *FGFR3, PIK3CA, HRAS, TP53, RB1*, and *PTEN* [4]. However, these genetic events alone do not fully account for the marked biological diversity, phenotypic plasticity, therapeutic resistance, and variable clinical behavior observed across bladder tumors. Increasing evidence indicates that these features are also shaped by epigenetic dysregulation, which influences gene expression without altering the underlying DNA sequence.

Epigenetic regulation comprises several interconnected mechanisms, including chromatin remodeling, histone modifications, DNA methylation, chromatin accessibility, enhancer–promoter communication, and higher-order chromatin organization [5]. In normal urothelial cells, these mechanisms coordinate lineage-specific transcription, differentiation, and responses to environmental stress [6]. In UBC, however, this regulatory network is frequently disrupted by mutations or aberrant activity in chromatin remodelers and histone-modifying enzymes, including ARID1A, KMT2C, KMT2D, EP300, CREBBP, KDM6A, EZH2, DNMTs, and HDACs [7]. These alterations reshape chromatin architecture, alter enhancer activity, silence tumor suppressor programs, sustain oncogenic transcription, and promote adaptation to therapeutic pressure.

Accumulating evidence indicates that epigenetic dysregulation can contribute functionally to selected aspects of UBC biology rather than representing only a downstream consequence of malignant transformation. Functional evidence is strongest for specific regulators and molecular contexts, whereas many reported epigenetic abnormalities remain associative. Altered chromatin states have been implicated in transcriptional plasticity, intratumoral heterogeneity, immune regulation, and treatment response. Because some epigenetic states are pharmacologically modifiable, these observations have generated interest in EZH2, HDAC, DNMT, BET, and related epigenetic pathways. However, the therapeutic relevance of individual alterations varies substantially, and most proposed epigenetic vulnerabilities in UBC remain preclinical or investigational [8].

In parallel, technological advances in single-cell epigenomics, spatial profiling, liquid biopsy, and AI-enabled multi-omics integration are transforming the field. These approaches enable increasingly precise mapping of chromatin states, regulatory dependencies, and tumor microenvironment interactions, thereby improving biomarker discovery and therapeutic stratification [9]. In UBC, where disease monitoring is clinically important and urine-based testing is particularly attractive, the development of epigenetic biomarkers also represents an active area of translational investigation [10]. The major epigenetic mechanisms discussed in this review, including chromatin remodeling, histone modifications, DNA methylation, and their downstream transcriptional consequences, are summarized in Figure 1. The broader transition from normal urothelial epigenetic organization to malignant enhancer-driven transcriptional reprogramming is illustrated in Figure 2, while the translational and technological workflow for epigenetic research and biomarker development in UBC is outlined in Figure 3.

**Figure 1.**
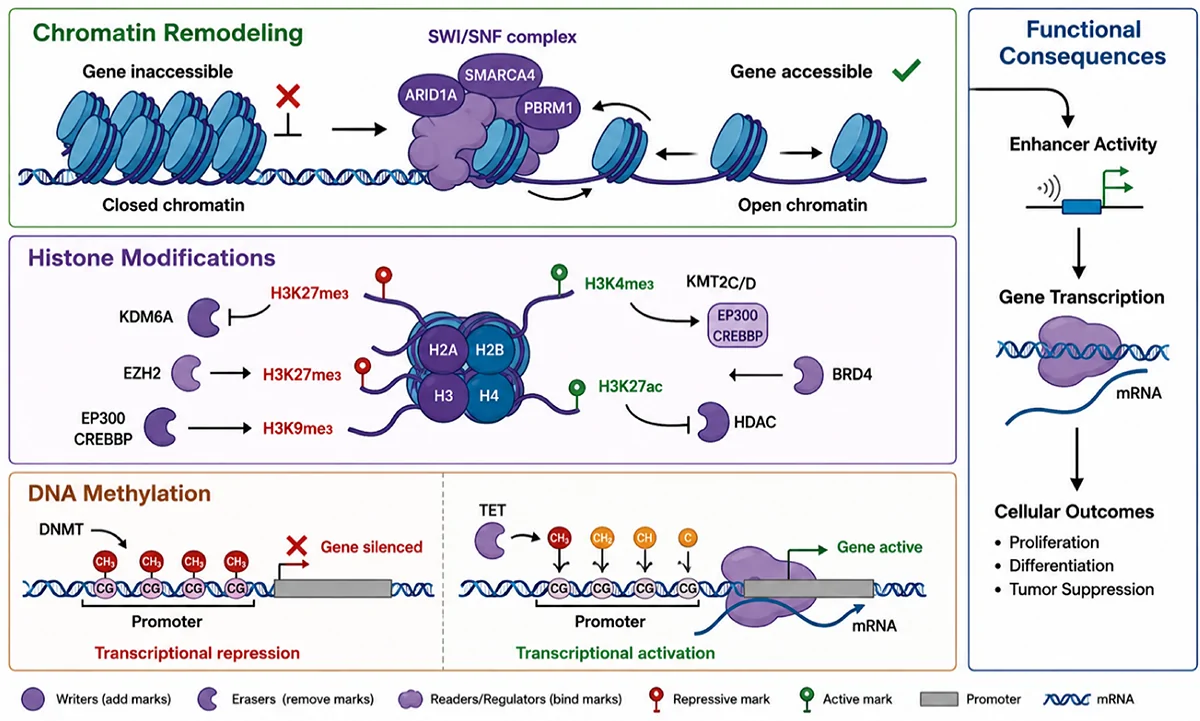
Fundamental epigenetic mechanisms in UBC [11].

**Figure 2.**
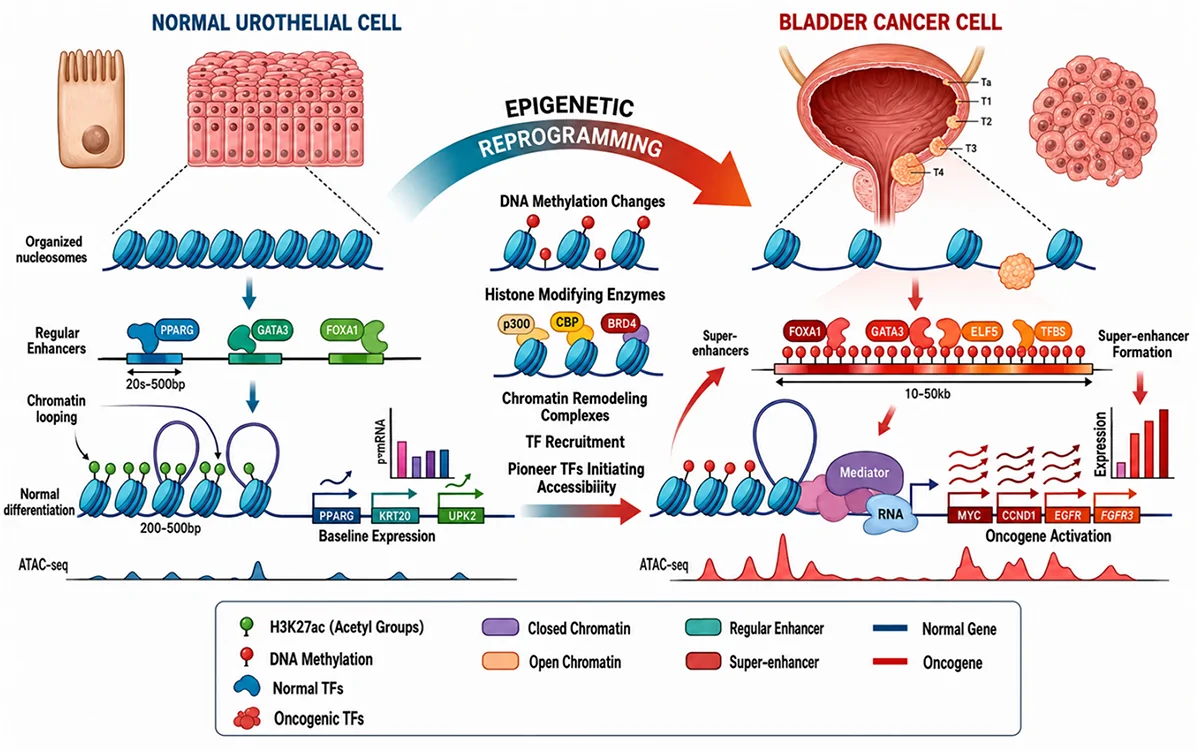
Epigenetic reprogramming from normal urothelium to bladder cancer [12].

**Figure 3.**
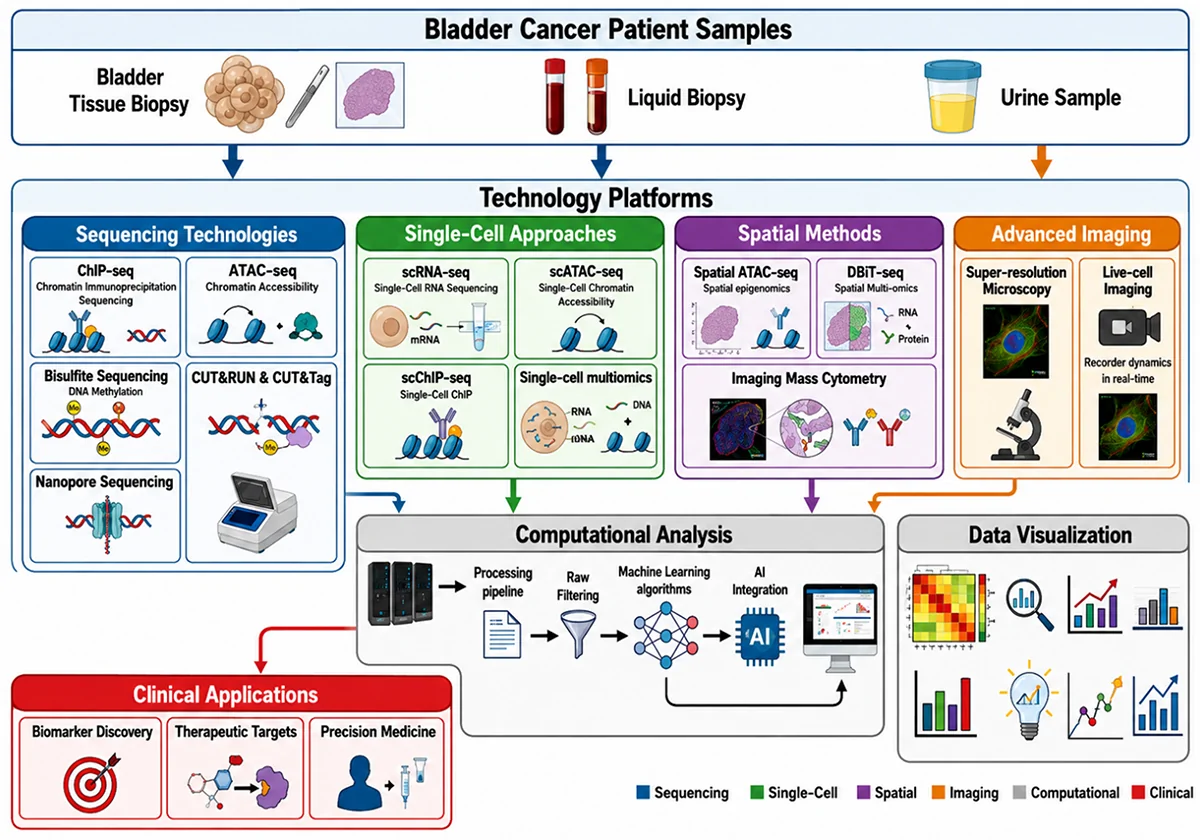
Integrative workflow for epigenetic research and translational development in urothelial bladder cancer.

Several previous reviews have described chromatin-regulatory alterations, DNA methylation abnormalities, epigenetic biomarkers, or experimental epigenetic therapies in bladder cancer. However, cataloguing recurrent alterations does not establish whether an epigenetic event is functionally causal, clinically measurable, or therapeutically actionable. The specific aim of the present review is therefore to evaluate UBC epigenetics across three interconnected levels of evidence: molecular mechanism, biomarker utility, and therapeutic validation. For each major epigenetic pathway, we distinguish recurrent molecular associations from experimentally demonstrated dependencies and further distinguish preclinical therapeutic rationale from evidence obtained in patients. Particular attention is given to areas in which these levels of evidence remain disconnected, including KDM6A/EZH2 dependency, liquid-biopsy methylation biomarkers, enhancer-associated vulnerabilities, and immuno-epigenetic combinations. This mechanism-to-biomarker-to-therapy framework is intended not simply to summarize the field, but to identify which epigenetic alterations have credible translational support and which remain hypotheses requiring functional or prospective clinical validation.

This review synthesizes current knowledge on epigenetic reprogramming in urothelial bladder cancer, with emphasis on chromatin remodeling, histone dysregulation, DNA methylation, enhancer and super-enhancer remodeling, tumor–immune interactions, biomarker development, and therapeutic targeting. It further highlights emerging technologies and translational opportunities that may support precision oncology approaches in UBC.

## Search strategy and study selection

This article was prepared as a structured narrative review. Literature searches were conducted in PubMed, Scopus, and Web of Science from January 2014 to June 2026, with older landmark mechanistic studies included only when necessary to explain foundational epigenetic concepts. Search terms included combinations of ‘urothelial bladder cancer,’ ‘bladder cancer,’ ‘epigenetics,’ ‘chromatin remodeling,’ ‘SWI/SNF,’ ‘ARID1A,’ ‘KDM6A,’ ‘EZH2,’ ‘histone modification,’ ‘DNA methylation,’ ‘enhancer,’ ‘super-enhancer,’ ‘liquid biopsy,’ ‘urine methylation,’ ‘immune evasion,’ ‘single-cell epigenomics,’ ‘spatial transcriptomics,’ ‘BET inhibitor,’ ‘HDAC inhibitor,’ ‘DNMT inhibitor,’ and ‘epigenetic therapy.’

Priority was given to peer-reviewed original studies, clinical trials, translational studies, high-quality systematic reviews, and major narrative reviews directly relevant to urothelial bladder cancer. Studies were included if they addressed epigenetic mechanisms in UBC, linked epigenetic changes to tumor heterogeneity or therapeutic resistance, evaluated epigenetic biomarkers in tissue, urine, or plasma, or investigated epigenetic therapeutic strategies. Publications were excluded if they focused mainly on non-urothelial cancers without clear relevance to UBC, were not peer reviewed, duplicated findings already covered by higher-quality studies, lacked mechanistic or clinical relevance, or presented unsupported claims. Preprints were not used as primary evidence unless clearly identified as preliminary and supported by peer-reviewed literature.

## Chromatin remodeling

Chromatin remodeling is a fundamental determinant of transcriptional control in urothelial cells because it regulates nucleosome positioning and the accessibility of DNA to transcription factors, coactivators, and DNA repair machinery [7]. In UBC, disruption of chromatin remodeling complexes represents one of the most biologically important epigenetic events, with direct consequences for lineage identity, enhancer function, and therapeutic response. Key chromatin remodeling abnormalities and their functional and therapeutic implications in UBC are summarized in Table 1 and conceptually illustrated in the top panel of Figure 1.

**Table 1.** UBC-specific evidence for chromatin-regulatory alterations and transcriptional plasticity.

Chromatin regulator / pathway	UBC-specific alteration or evidence	Mechanistic consequence supported by evidence	Translational / therapeutic interpretation	Evidence level	Supporting references
ARID1A / SWI/SNF	Recurrent loss-of-function alterations and loss of ARID1A expression occur in UBC. In NMIBC, ARID1A alterations have been associated with increased recurrence risk following BCG, although predictive versus prognostic significance remains unresolved.	ARID1A loss disrupts SWI/SNF-dependent chromatin regulation and can create context-specific signaling dependencies. Experimental ARID1A-deficient bladder cancer models demonstrate dependency on PI3K signaling.	ARID1A should not be presented as an established biomarker of sensitivity to EZH2 inhibitor monotherapy. One UBC study found no enhanced sensitivity to EZH2 inhibition, whereas subsequent preclinical work demonstrated synergistic activity from combined EZH2 and PI3K inhibition.	Human genomic / clinical association plus preclinical functional evidence. Not clinically validated as an EZH2-response biomarker.	[13–16]
KMT2C / KMT2D	KMT2C and KMT2D are recurrently altered chromatin regulators in bladder cancer. Urothelial loss of Kmt2c or Kmt2d in genetically engineered models impairs differentiation and increases susceptibility to oncogenic transformation.	Loss of KMT2C/KMT2D perturbs enhancer-associated chromatin regulation and redistributes KMT2A-menin activity toward bivalent promoters, producing a urothelial state more permissive to tumorigenesis.	Provides strong biological evidence that KMT2C/KMT2D loss contributes functionally to urothelial tumor susceptibility. Sensitivity to BET inhibitors or other enhancer-directed drugs has not been clinically established and should not be stated as an accepted therapeutic vulnerability.	Human genomic evidence plus strong genetically engineered preclinical evidence.	[15,17]
Chromatin-regulator alterations collectively	UBC shows a high burden of recurrent alterations involving multiple chromatin-regulatory genes, including KDM6A, KMT2-family genes, CREBBP, EP300 and ARID1A.	These alterations affect distinct but interacting processes including chromatin accessibility, enhancer regulation, histone modification and transcriptional control.	The high prevalence of chromatin-regulator alterations supports molecular stratification and functional investigation, but the presence of a chromatin-regulator mutation alone should not be equated with therapeutic actionability.	Human tumor genomic evidence.	[15]

Recurrent disruption of chromatin-regulatory machinery is a characteristic molecular feature of UBC. Among individual regulators, ARID1A and KMT2C/KMT2D have comparatively well-developed UBC-specific evidence linking recurrent alteration to functional changes in chromatin regulation. The biological consequences are regulator- and context-specific, however, and recurrent mutation alone should not be interpreted as evidence of a common downstream phenotype or therapeutic dependency.

ARID1A is among the recurrently altered SWI/SNF components in urothelial bladder cancer, and its loss has been associated with changes in chromatin regulation, DNA-damage responses, and treatment-related phenotypes [13]. However, the therapeutic consequences of ARID1A deficiency appear to be context dependent. In UBC models, ARID1A loss has not consistently predicted intrinsic sensitivity to EZH2 inhibition, whereas other experimental work has demonstrated enhanced vulnerability when EZH2 inhibition is combined with disruption of compensatory pathways such as PI3K signaling [14]. ARID1A status should therefore be regarded as a candidate molecular stratification feature rather than an established predictive biomarker for EZH2-directed therapy.

KMT2C and KMT2D are recurrently altered members of an enhancer-regulatory complex that also includes KDM6A. Recent functional evidence supports a direct role for KMT2C/KMT2D loss in urothelial tumor susceptibility rather than merely an association with established cancer. In genetically engineered models, urothelial loss of Kmt2c or Kmt2d impaired differentiation, enhanced responses to proliferative and inflammatory stimuli, and increased susceptibility to oncogenic transformation [17]. These findings provide functional evidence that disruption of enhancer-associated chromatin regulation can create a permissive state for urothelial tumorigenesis. However, whether KMT2C/KMT2D alterations predict sensitivity to BET inhibitors or other enhancer-directed therapies in patients with UBC remains unproven and should be considered a preclinical hypothesis rather than an established therapeutic vulnerability.

Taken together, recurrent alterations of chromatin-regulatory genes are a prominent molecular feature of UBC, but the biological and therapeutic consequences differ substantially among individual regulators. Experimental evidence supports functional roles for selected alterations rather than a uniform effect of chromatin-remodeling dysfunction. For example, urothelial loss of KMT2C or KMT2D impairs differentiation and increases susceptibility to oncogenic transformation, providing direct evidence that disruption of enhancer-associated chromatin regulation can contribute to urothelial tumorigenesis [17]. By contrast, the therapeutic implications of ARID1A loss are more context dependent. ARID1A deficiency was not associated with increased sensitivity to EZH2 inhibition in one UBC study, whereas subsequent work identified PI3K pathway dependency and synergistic activity from combined EZH2 and PI3K inhibition in ARID1A-deficient bladder cancer models [13,14].

These observations illustrate why recurrent alteration should not be equated automatically with therapeutic actionability. Chromatin-regulator mutations may help define biologically distinct UBC subsets and generate testable therapeutic hypotheses, but predictive value must be demonstrated separately for each alteration and intervention. Table 1 therefore summarizes UBC-specific evidence for selected chromatin-regulatory abnormalities while distinguishing mechanistic findings from candidate therapeutic implications that remain preclinical or clinically unvalidated.

This schematic summarizes the major epigenetic mechanisms involved in urothelial bladder cancer (UBC), including aberrant chromatin remodeling, dysregulated histone modifications, and altered DNA methylation. In the top panel, disruption of chromatin remodeling complexes such as SWI/SNF alters nucleosome positioning and chromatin accessibility, thereby influencing enhancer activity and transcriptional output. In the middle panel, changes in histone-modifying enzymes, including EZH2, KDM6A, EP300, CREBBP, BRD4, and HDACs, reshape activating and repressive histone marks, resulting in oncogenic transcriptional reprogramming. In the lower panel, promoter hypermethylation contributes to tumor suppressor gene silencing, whereas demethylation promotes transcriptional reactivation. Together, these mechanisms converge on enhancer function, gene transcription, and downstream cellular outcomes including proliferation, differentiation blockade, tumor progression, and therapeutic resistance. The figure also highlights clinically relevant therapy targets, including EZH2, HDAC, BET/BRD4, and DNMT-associated pathways.

## Histone modifications

Histone modifications regulate chromatin accessibility and transcription through the addition, removal, or recognition of covalent marks on histone proteins. In UBC, recurrent alterations involving histone-modifying enzymes are well documented, but the functional evidence and translational maturity of individual pathways are heterogeneous. Particular attention has focused on the opposing regulation of H3K27 methylation by KDM6A and EZH2, recurrent alterations of the acetyltransferases EP300 and CREBBP, and experimental targeting of HDAC and BET-dependent transcriptional programs. Rather than treating these abnormalities as equivalent therapeutic targets, the following section distinguishes direct UBC-specific mechanistic evidence from preclinical therapeutic hypotheses and clinical observations. The strength of evidence for the principal histone-regulatory pathways is summarized in Table 2.

**Table 2.** Histone-regulatory abnormalities in UBC: mechanistic evidence and therapeutic interpretation.

Epigenetic regulator / pathway	UBC-specific finding	Functional consequence	Therapeutic interpretation	Evidence level	Supporting references
KDM6A	KDM6A is recurrently inactivated in bladder cancer. Experimental loss of KDM6A increases PRC2-associated transcriptional repression and disrupts normal urothelial differentiation.	KDM6A loss alters the balance of H3K27 methylation, suppresses differentiation programs and can establish a proliferative epigenetic state.	KDM6A loss provides a mechanistic rationale for EZH2 inhibition. However, KDM6A mutation is not yet a clinically validated predictive biomarker for EZH2 inhibitor therapy in UBC.	Human genomic plus strong mechanistic preclinical evidence.	[18,19]
EZH2 / PRC2	EZH2-mediated H3K27 trimethylation provides an opposing regulatory activity to KDM6A. Pharmacological EZH2 inhibition has shown antitumor effects in bladder cancer models, particularly in KDM6A-deficient settings.	EZH2 inhibition can reduce repressive H3K27 methylation, alter differentiation-associated transcription and promote immune-related responses. In MIBC models, EZH2 inhibition induced NK-cell-associated differentiation and tumor-cell death.	One of the better-supported preclinical epigenetic vulnerabilities in UBC. Translation is now being investigated clinically, but clinical efficacy and predictive biomarkers remain unconfirmed.	Strong preclinical mechanistic evidence plus early clinical investigation.	[18,20]
EP300 / CREBBP	Recurrent inactivating alterations of the histone acetyltransferases EP300 and CREBBP occur in UBC. Functional studies have linked these alterations to altered acetyltransferase-dependent transcriptional programs.	Reduced acetyltransferase activity can alter enhancer and promoter acetylation and transcriptional regulation.	EP300/CREBBP alterations have been investigated as biomarkers for HDAC-directed treatment, but a biomarker-selected mocetinostat phase II study demonstrated limited activity. Therefore, EP300/CREBBP alteration should not be described as a validated predictive biomarker.	Human genomic / translational evidence plus biomarker-selected phase II clinical evidence.	[15,21,22]
HDAC1/3 and class I HDAC activity	HDAC inhibition has been investigated as a method of modifying UBC transcriptional and immune states rather than because of a single recurrent HDAC mutation. Entinostat produced immune editing and enhanced antitumor immune activity in preclinical urothelial models.	HDAC inhibition can alter tumor-cell transcription, antigenicity and immune microenvironment interactions.	Clinical trials of HDAC inhibition in UBC have produced mixed or negative results. Vorinostat monotherapy was ineffective and toxic, while combinations with checkpoint inhibition remain exploratory.	Preclinical mechanistic plus early clinical evidence. No established UBC indication.	[23–25]
BRD4 / BET proteins	BRD4 participates in oncogenic transcription in bladder cancer. Experimental studies showed BRD4-dependent regulation of c-MYC/EZH2 signaling and antiproliferative effects following BET inhibition. Combined BET and class I HDAC inhibition produced synergistic apoptosis in urothelial carcinoma cell lines.	BET inhibition disrupts acetyl-lysine-dependent transcriptional regulation and can suppress oncogenic transcriptional programs.	Evidence in UBC remains preclinical. BET dependency should not be described as clinically actionable or “promising” without qualification. No validated UBC biomarker for BET inhibitor selection has been established.	Preclinical mechanistic evidence only.	[26,27]

A major feature of UBC is the recurrent alteration of enzymes that write, erase, or interpret histone marks. Recurrent loss-of-function alterations involving the histone acetyltransferases EP300 and CREBBP have been identified in UBC [28]. These alterations provide evidence that disruption of acetyltransferase-dependent transcription contributes to UBC biology; however, the downstream consequences are context dependent and should not be inferred uniformly from mutation status alone. Importantly, the presence of EP300 or CREBBP alterations has not been validated as a reliable predictor of response to HDAC-directed therapy, as illustrated by the limited activity observed in a biomarker-selected clinical study of mocetinostat [21]. In practical terms, the sequence of events can be described as loss of acetyltransferase activity→ reduced enhancer/promoter acetylation→ repression of tumor-suppressive transcriptional programs→ increased proliferation and survival.

At the same time, overactivity of repressive histone methylation pathways also contributes to tumor progression. EZH2, the catalytic subunit of the polycomb repressive complex 2, mediates H3K27 trimethylation, a modification associated with transcriptional silencing. In UBC, heightened EZH2 activity can suppress genes involved in differentiation, cell-cycle control, inflammatory signaling, and antigen presentation [29]. The result is a more dedifferentiated, aggressive, and immune-evasive phenotype. This effect may be further amplified by loss of KDM6A, a demethylase that counterbalances repressive H3K27 methylation. When KDM6A function is lost, removal of repressive histone marks is impaired, enhancer flexibility increases, and oncogenic signaling is reinforced.

The relationship between KDM6A loss and EZH2 activity provides one of the strongest experimentally supported epigenetic dependencies in bladder cancer. Ler et al. demonstrated that loss of KDM6A amplifies PRC2-mediated transcriptional repression and that KDM6A-deficient bladder cancer models can be targeted through pharmacological inhibition of EZH2 [18]. Subsequent work in muscle-invasive bladder cancer showed that EZH2 inhibition can induce immune-associated transcriptional responses, promote natural killer cell-mediated differentiation and tumor-cell death, and enhance antitumor effects when combined with cisplatin [20]. More recent functional studies have further shown that KDM6A loss disrupts normal urothelial differentiation and promotes a proliferative epigenetic state [19]. Collectively, these studies provide a mechanistic rationale for EZH2-directed therapy in KDM6A-altered UBC. Nevertheless, this evidence remains predominantly preclinical, and KDM6A mutation has not yet been established as a clinically validated predictive biomarker for EZH2 inhibitor treatment.

These histone-based alterations do not operate independently. Instead, they interact with chromatin remodeling defects and DNA methylation changes to define regulatory landscapes that sustain malignant transcriptional states [30]. Of particular importance is the formation and maintenance of super-enhancers, which are highly active regulatory hubs enriched for acetylation-associated marks such as H3K27ac and occupied by cofactors including BRD4. In aggressive UBC, dysregulated acetylation and methylation can shift the enhancer repertoire toward one that favors invasion, angiogenesis, metabolic adaptation, and therapeutic resistance.

The evidence for other histone-directed strategies is less advanced. HDAC inhibition can modify transcriptional and immune states experimentally, but clinical studies in urothelial carcinoma have shown limited efficacy or unfavorable toxicity in some settings. Similarly, BET/BRD4 inhibition suppresses oncogenic transcription in bladder cancer models, but evidence remains predominantly preclinical. Accordingly, terms such as ‘therapeutic target’ or ‘sensitivity’ should be interpreted according to the level of evidence and should not imply established clinical benefit. Table 2 summarizes these pathways together with the distinction between mechanistic, preclinical, and clinical evidence.

## DNA methylation

DNA methylation is among the most extensively investigated epigenetic mechanisms in bladder cancer and has relevance at both biological and biomarker levels. Aberrant promoter hypermethylation can contribute to transcriptional repression, whereas broader methylation changes may reflect tumor-associated alterations in cell state and genomic regulation. However, evidence should be distinguished between mechanistic observations in tumor tissue and the clinical performance of individual methylation assays. In UBC, the most mature translational application of DNA methylation is currently its detection in urine, while plasma cell-free DNA methylation represents a less developed but potentially complementary approach. Figure 1 illustrates the general transcriptional consequences of promoter methylation, whereas Table 3 focuses specifically on methylation-based liquid-biopsy assays for which UBC-specific clinical data are available.

**Table 3.** UBC DNA methylation biomarkers evaluated in urine and plasma.

Biomarker / assay	Sample	Intended clinical application	Key UBC-specific evidence	Clinical interpretation	Evidence level	Supporting reference
PENK DNA methylation test	Urine	Detection of bladder cancer in patients with hematuria	In a prospective multicenter study of 1,099 participants, the assay showed 89.2% sensitivity and 87.8% specificity for high-grade or invasive bladder cancer. NPV was 97.6%, whereas PPV was 61.3%.	Strong evidence for non-invasive detection in a hematuria population, particularly for excluding high-grade/invasive disease. The suboptimal PPV means it should not be presented as a replacement for cystoscopy or standard diagnostic pathways.	Prospective multicenter clinical validation.	[31]
ONECUT2, SOX1, TWIST1 and NKX2-4 methylation panel	Urine	Non-invasive diagnosis of bladder urothelial carcinoma	In 319 urine samples from individuals with suspected bladder urothelial carcinoma, the four-gene panel achieved an AUC of 0.971 and accuracy of 92.39%.	Strong biomarker-development performance, but external prospective validation is still needed before routine clinical implementation.	Clinical biomarker development with internal validation.	[32]
Bladder EpiCheck methylation assay	Voided urine	Surveillance for NMIBC recurrence	In a prospective blinded North American study, 449 evaluable patients had overall sensitivity of 67% and specificity of 84%. For high-grade recurrence, sensitivity was 77% and NPV 95%.	Potential adjunct for NMIBC surveillance, particularly for ruling out high-grade recurrence. It should be described as complementary to cystoscopy rather than a universal replacement.	Prospective multicenter clinical evidence.	[33]
Bladder EpiCheck before and after BCG	Urine	Prediction of recurrence and BCG failure	In a prospective cohort of 65 patients, post-BCG EpiCheck status and changes in EpiScore were associated with recurrence and BCG failure. The study specifically states that larger independent validation is required.	Interesting treatment-monitoring application, but currently exploratory because of the small cohort and lack of external validation.	Prospective exploratory clinical evidence.	[34]
Genome-wide cfDNA methylation signatures, SWOG S1314	Plasma	Prediction of response to neoadjuvant chemotherapy in MIBC	Exploratory analysis of SWOG S1314 demonstrated that plasma cell-free DNA methylation can be profiled to generate signatures associated with response to neoadjuvant chemotherapy.	Provides important proof-of-concept for plasma epigenetic liquid biopsy, but it remains an investigational predictive approach and requires prospective validation in independent cohorts.	Exploratory clinical / translational evidence from a prospective trial cohort.	[35]

Promoter hypermethylation commonly affects tumor suppressor genes and regulatory pathways involved in cell-cycle control, apoptosis, DNA repair, adhesion, and immune surveillance. When CpG-rich promoter regions become aberrantly methylated, transcription is repressed, often in a stable and heritable manner. In bladder cancer, this can lead to silencing of genes such as CDKN2A and RASSF1A, thereby removing constraints on proliferation and survival [36]. These observations support a model in which promoter hypermethylation can contribute to transcriptional silencing of tumor-suppressive programs, although the extent to which individual methylation events function as causal drivers rather than markers of tumor state varies among loci and requires gene-specific functional validation [37].

In contrast, global hypomethylation contributes to tumor biology by destabilizing the genome and derepressing normally silenced sequences. This process may activate proto-oncogenic pathways, alter chromosomal integrity, and reactivate repetitive or transposable elements. Such changes can intensify genomic instability and accelerate tumor evolution. Although promoter hypermethylation and global hypomethylation are often discussed separately, they frequently coexist in the same tumor and cooperate with histone modifications and chromatin remodeling defects to reinforce malignant transcriptional programs [38].

### Epigenetic biomarkers in liquid biopsy

Liquid-biopsy analysis represents one of the most clinically developed applications of epigenetics in UBC, although the evidence differs substantially between urine and blood. Urine is particularly attractive because it is in direct contact with urothelial tumors and may contain exfoliated tumor cells and tumor-derived cell-free DNA. Multiple methylation-based assays have therefore been evaluated for diagnosis and surveillance, with recent prospective studies demonstrating clinically relevant sensitivity for high-grade or invasive disease and for detection of recurrent NMIBC [31,33]. Nevertheless, performance varies according to the methylation targets, assay platform, patient population, tumor stage and grade, and intended clinical use.

The distinction between diagnostic performance and clinical utility is important. High sensitivity or negative predictive value does not by itself demonstrate that a urine methylation assay can safely replace cystoscopy, cytology, or standard hematuria evaluation. Some assays may ultimately function as adjuncts that reduce unnecessary invasive procedures, whereas others may prove more useful for recurrence surveillance or treatment monitoring. Prospective comparative studies and demonstration that biomarker-guided management is non-inferior or superior to existing pathways are required before broad substitution of established surveillance methods.

Plasma methylation biomarkers are less extensively studied in UBC. Unlike urine, plasma cell-free DNA contains molecular material released from multiple tissues, and the tumor-derived fraction may be low in localized bladder cancer. Nevertheless, analysis of plasma cfDNA methylation in patients enrolled in the prospective SWOG S1314 neoadjuvant chemotherapy trial provided proof of concept that methylation profiles can contain information associated with treatment response in muscle-invasive bladder cancer [35]. This finding supports further investigation of plasma methylation for treatment-response prediction and longitudinal monitoring but does not establish a clinically validated predictive assay.

Urine and plasma should therefore be considered complementary rather than interchangeable sources of epigenetic information. Current evidence is substantially more developed for urinary methylation-based detection and surveillance, whereas plasma cfDNA methylation remains exploratory in UBC. Table 3 summarizes representative UBC studies and reports their clinical setting, principal findings, and level of evidence rather than presenting liquid-biopsy methylation as an established clinical tool.

Taken together, methylation-based liquid biopsy illustrates both the opportunity and the current limitations of epigenetic biomarker translation in UBC. Several urine assays have progressed beyond biomarker discovery into prospective clinical evaluation, whereas evidence for plasma methylation remains comparatively early. Future studies should emphasize independent validation, standardized pre-analytical and analytical methods, predefined clinical-use scenarios, direct comparison with existing diagnostic pathways, and demonstration that testing changes management or improves patient outcomes.

## Enhancer reprogramming

Enhancers are cis-regulatory elements that coordinate cell-state-specific transcription through interactions with transcription factors, chromatin regulators, and transcriptional coactivators [39]. Super-enhancers are larger clusters of highly active enhancers that drive especially strong transcription of genes involved in cell identity and disease-specific dependencies. UBC-specific epigenomic studies have identified subtype-associated enhancer landscapes and transcription-factor networks, supporting an association between enhancer reprogramming and distinct urothelial cancer cell states [40]. The extent to which individual enhancer changes independently drive progression or predict therapeutic response remains incompletely defined. The shift from normal enhancer organization to super-enhancer-driven oncogenic transcription is illustrated in Figure 2.

In normal urothelium, enhancer landscapes support differentiation and epithelial homeostasis through tightly regulated transcription factor networks. In bladder cancer, however, mutations in chromatin regulators such as *ARID1A, KMT2C, KMT2D, EP300*, and *CREBBP*, together with altered chromatin accessibility and histone marking, reshape this enhancer architecture [7]. The result is a shift from lineage-constrained regulation toward enhancer programs that sustain oncogenic growth, stress adaptation, and phenotypic flexibility.

This process is especially important in aggressive MIBC, where enhancer remodeling can support persistent expression of genes involved in proliferation, angiogenesis, invasion, and survival, including pathways associated with MYC, FGFR3, and VEGFA [41]. In practical terms, the mechanistic sequence is chromatin or histone regulator dysfunction→ altered enhancer accessibility and occupancy→ activation of tumor-promoting transcriptional networks→ invasion, progression, and treatment resistance [42].

Super-enhancers represent highly active regulatory regions capable of sustaining high-output transcriptional programs. UBC-specific epigenomic studies support subtype-associated enhancer reprogramming, although the degree to which individual tumors are functionally dependent on particular super-enhancer programs remains incompletely established. Because BET proteins participate in acetylation-dependent enhancer transcription, experimental bladder cancer studies have investigated BET inhibition as a strategy to disrupt oncogenic transcriptional programs [26,27]. These findings remain preclinical, and super-enhancer dependence has not been validated as a clinical biomarker for BET-directed therapy in UBC.

Enhancer reprogramming also helps explain why UBC exhibits such marked transcriptional plasticity. By reconfiguring the regulatory elements that determine cell-state identity, tumors can shift dynamically in response to hypoxia, therapy, and microenvironmental stress. This may facilitate resistance to chemotherapy, targeted therapy, and immunotherapy. Accordingly, enhancer biology is not simply a mechanistic detail; it is central to understanding how tumors change over time and why static molecular classifications may be insufficient.

## Tumor-immune interactions

Epigenetic dysregulation can influence tumor-immune interactions in UBC through effects on antigen presentation, inflammatory signaling, chemokine expression, and other immune-regulatory programs [29]. In this context, the epigenome functions as a regulatory interface between tumor-intrinsic biology and the immune microenvironment.

Epigenetic regulation of antitumor immunity in UBC is highly context dependent and should not be interpreted as uniformly immunosuppressive. Altered chromatin accessibility can influence antigen-processing pathways, interferon-responsive transcription, chemokine expression, and immune-cell recruitment [43]. However, the effect of individual chromatin regulators may differ. In metastatic urothelial carcinoma, ARID1A mutation combined with high CXCL13 expression was associated with improved outcomes following immune checkpoint therapy, while experimental ARID1A knockdown increased checkpoint sensitivity in a bladder cancer model [44]. These findings argue against a generalized assumption that ARID1A loss necessarily reduces tumor immune visibility. By contrast, repressive chromatin states involving PRC2/EZH2 provide a mechanistically supported route through which immune-related transcription can be suppressed, providing a rationale for investigating immuno-epigenetic combinations. As summarized schematically in Figure 2, epigenetic reprogramming not only sustains oncogenic transcription but also contributes to immune evasion through repression of antigen presentation and inflammatory gene programs.

Epigenetic repression of immune-regulatory loci may reduce antigen presentation, inflammatory signaling, T-cell recruitment, and immune recognition, thereby contributing to immune evasion and checkpoint resistance. Epigenetic regulation may also affect the expression of checkpoint ligands and co-regulatory molecules, linking chromatin state to immune escape more broadly. In this setting, the tumor epigenome functions as both a barrier to immune recognition and a therapeutic opportunity. Pharmacologic inhibition of EZH2 or HDACs may reopen silenced regulatory regions, increase antigen-presentation machinery, enhance chemokine release, and improve immune-cell infiltration. These observations suggest that epigenomic profiling may generate candidate biomarkers of immune-refractory states and provide hypotheses for biomarker-informed immuno-epigenetic combinations; prospective clinical validation remains required.

His figure illustrates the transition from a normal urothelial epigenetic landscape to a malignant bladder cancer regulatory state. In normal urothelial cells, organized nucleosomes, lineage-associated transcription factors, regular enhancers, and controlled chromatin looping maintain differentiation-linked transcriptional programs. In bladder cancer cells, epigenetic reprogramming driven by chromatin regulator mutations, histone modification imbalance, altered DNA methylation, nucleosome displacement, and super-enhancer formation results in aberrant transcription factor recruitment, oncogene activation, transcriptional plasticity, and aggressive tumor behavior. This cause-to-mechanism-to-outcome model links epigenetic dysregulation to invasion, immune evasion, and disease progression, while emphasizing candidate enhancer-dependent therapeutic vulnerabilities.

## Therapeutic targeting

The reversibility of epigenetic regulation provides a biological rationale for therapeutic intervention, but reversibility should not be equated with drug sensitivity or clinical actionability. Evidence for epigenetic therapy in UBC ranges from molecular dependencies demonstrated in experimental models to early-phase clinical trials, and the strength of evidence differs considerably among EZH2, HDAC, DNMT, and BET-directed approaches. At present, epigenetic therapies should therefore be discussed according to whether support is preclinical, exploratory clinical, or derived from trials demonstrating meaningful patient benefit.

EZH2 inhibition represents one of the better-supported UBC-specific preclinical strategies. KDM6A loss can increase PRC2-associated transcriptional repression, and pharmacological inhibition of EZH2 has demonstrated antitumor activity in KDM6A-deficient bladder cancer models [18]. Further work showed that EZH2 inhibition can induce differentiation-associated and immune responses and can augment cisplatin activity in selected MIBC models [20]. These findings provide a mechanistic rationale for clinical investigation. However, neither KDM6A nor other chromatin-regulator alterations have yet been established as prospectively validated predictive biomarkers for EZH2 inhibition in UBC.

The relationship between ARID1A loss and EZH2 sensitivity is particularly instructive because UBC-specific studies have produced context-dependent findings. One study found that ARID1A deficiency did not confer enhanced sensitivity to EZH2 inhibition [13], whereas later experimental work demonstrated PI3K pathway dependency and synergistic activity from combined PI3K and EZH2 inhibition in ARID1A-deficient bladder cancer [14]. ARID1A alteration should therefore not be described as an established biomarker for EZH2 inhibitor monotherapy.

Clinical investigation of HDAC inhibitors has similarly demonstrated that mechanistic rationale does not necessarily translate into useful antitumor activity. Vorinostat monotherapy showed limited efficacy and significant toxicity in previously treated recurrent or metastatic urothelial carcinoma [24]. A biomarker-selected phase II study of mocetinostat in tumors harboring CREBBP and/or EP300 alterations also demonstrated limited activity, indicating that these acetyltransferase alterations were insufficient to establish a clinically useful predictive biomarker for HDAC inhibition [21]. Combination of vorinostat with pembrolizumab has demonstrated feasibility in an early-phase study that included urothelial carcinoma, but the mixed tumor population and limited activity prevent conclusions regarding UBC-specific efficacy [25].

DNMT-directed clinical studies provide another example of the gap between pharmacodynamic activity and clinical benefit. In the SPIRE study, guadecitabine was combined with gemcitabine and cisplatin and produced measurable pharmacodynamic demethylation, but treatment was associated with substantial hematologic toxicity and required granulocyte colony-stimulating factor prophylaxis [45]. More importantly, a subsequent phase II study of guadecitabine plus atezolizumab in metastatic urothelial carcinoma progressing after immune checkpoint inhibition reported no RECIST objective responses and did not demonstrate the anticipated tumor-intrinsic demethylation effect [46]. These negative findings are important because they demonstrate that epigenetic modulation alone does not guarantee restoration of treatment sensitivity.

BET/BRD4 inhibition has a biologically plausible role in disrupting enhancer-associated transcriptional programs, and experimental bladder cancer studies have demonstrated suppression of oncogenic transcription and synergistic effects with class I HDAC inhibition [26,27]. However, this evidence remains preclinical. BET inhibitors should therefore not be described as clinically ‘promising’ in UBC without qualification, and no validated biomarker currently identifies a UBC population known to benefit clinically from BET-directed treatment.

### Limitations of epigenetic therapy

Several challenges limit the clinical translation of epigenetic therapy in UBC. First, many epigenetic agents modify broad transcriptional programs rather than a single tumor-specific dependency, increasing the potential for off-target effects and dose-limiting toxicity. Second, intratumoral heterogeneity may result in variable dependency on the targeted pathway even among tumors carrying the same molecular alteration. Third, epigenetic states are dynamic and may change under therapeutic pressure, enabling adaptive resistance. Fourth, recurrent mutations such as ARID1A or KDM6A alterations do not automatically establish continued functional dependency on a particular drug target. Finally, the limited number of biomarker-selected prospective trials makes it difficult to define which patients are most likely to benefit.

Future epigenetic trials in UBC should therefore incorporate predefined molecular selection strategies, pharmacodynamic confirmation of target modulation, longitudinal tissue or liquid-biopsy assessment, and clinically meaningful efficacy endpoints. Negative and terminated studies are equally important to this evaluation because they define the limits of biological hypotheses and prevent preclinical observations from being interpreted prematurely as clinically actionable treatment strategies. Table 4 summarizes representative preclinical and clinical studies according to the specific agent, molecular target, biomarker strategy, therapeutic setting, efficacy, toxicity, trial status, and overall level of evidence.

**Table 4.** Preclinical and clinical evaluation of epigenetic therapies in urothelial bladder cancer.

Agent / regimen	Epigenetic target	Setting / phase and registration	UBC population / model	Biomarker strategy	Major efficacy finding	Major toxicity / limitation	Current interpretation / evidence level	Supporting reference
GSK343, EZH2 inhibition	EZH2 / H3K27me3	Preclinical; no clinical trial phase	KDM6A-deficient bladder cancer models	KDM6A loss / PRC2-dependent repression	Pharmacological EZH2 inhibition preferentially suppressed KDM6A-deficient bladder cancer models, providing direct evidence of an epigenetic dependency.	Experimental models only; clinical therapeutic index and predictive accuracy of KDM6A status are unknown.	Strong preclinical mechanistic evidence. KDM6A is not yet a clinically validated predictive biomarker.	[18]
Tazemetostat + pembrolizumab	EZH2 + PD-1	Phase I/II, NCT03854474, ETCTN 10,183	Locally advanced or metastatic UC, including platinum-refractory and platinum-ineligible patients	Baseline EZH2, H3K27me3, histone-methylation-associated mutations and immune correlates	Early clinical activity has been reported, but mature phase II efficacy and biomarker-response results are not yet available.	Small pilot study; no randomized comparator; predictive biomarker hypothesis remains unvalidated.	Early clinical / exploratory. Active, not recruiting as of the June 2026 ClinicalTrials.gov update.	[47]
Vorinostat monotherapy	HDAC	Phase II, NCT00363883	Recurrent or metastatic urothelial carcinoma after prior platinum therapy	Correlative tissue, blood and gene-expression analyses; no validated selection biomarker	Fourteen patients were enrolled; the study did not demonstrate meaningful objective antitumor activity, with median progression-free survival approximately 1.1 months and overall survival 3.2 months.	Significant hematologic and other toxicity; unfavorable efficacy-to-toxicity profile.	Negative clinical study. Trial terminated early for futility. Important to include to balance the therapeutic discussion.	[24,48]
Mocetinostat monotherapy	Class I HDAC	Phase II, NCT02236195	Previously treated locally advanced or metastatic UC selected for CREBBP and/or EP300 inactivating alterations	CREBBP/EP300 loss-of-function selection	Seventeen patients entered stage 1; only nine were efficacy-evaluable and one partial response, 11%, was observed. Activity was insufficient to proceed to the next stage.	All patients experienced at least one adverse event; nausea and fatigue were common, and treatment interruptions and dose reductions were frequent.	Biomarker-selected phase II study with limited efficacy. CREBBP/EP300 status was not validated as a useful predictive biomarker for mocetinostat.	[21]
Vorinostat + pembrolizumab	HDAC + PD-1	Phase I/Ib, NCT02619253	Advanced renal or urothelial carcinoma, including checkpoint-naive and checkpoint-resistant cohorts	Pharmacodynamic and immune-modulatory analyses; no established UBC predictive biomarker	In mixed UC/RCC expansion cohorts, ORR was 8% in the checkpoint-naive cohort and 0% in the checkpoint-resistant cohort, with median PFS of 2.9 and 3.5 months, respectively.	Mixed tumor populations make UBC-specific efficacy difficult to determine. Resolved grade 3/4 toxicities included diarrhea, hypophosphatemia, acute kidney injury, anemia and hypothyroidism.	Feasibility demonstrated, but evidence for meaningful UBC-specific efficacy remains limited. Completed study.	[25]
Guadecitabine + gemcitabine/cisplatin, SPIRE	DNMT inhibition + chemotherapy	Phase I dose escalation with bladder cancer expansion, ISRCTN16332228	Solid malignancies with a neoadjuvant muscle-invasive bladder cancer expansion cohort	Pharmacodynamic DNA demethylation	A recommended dose of guadecitabine combined with gemcitabine/cisplatin was identified, demonstrating that epigenetic priming could be delivered with chemotherapy. The study was not sufficient to establish improved UBC efficacy.	Dose-limiting toxicity was predominantly myelosuppression. Grade 3 or greater neutropenia occurred in 76.5%, thrombocytopenia in 64.7%, leukopenia in 29.4%, and anemia in 29.4% of dose-escalation patients; G-CSF prophylaxis was required.	Early clinical feasibility with substantial hematologic toxicity. Not evidence of established therapeutic benefit.	[45]
Guadecitabine + atezolizumab	DNMT inhibition + PD-L1	Phase II, NCT03179943	Metastatic UC resistant or refractory to previous immune checkpoint inhibition	DNA methylation and immune pharmacodynamic analyses	No RECIST responses were observed. The anticipated tumor-intrinsic demethylation effect was also not demonstrated after treatment.	Absence of objective responses and failure to demonstrate the expected tumor demethylation effect substantially weaken the proposed therapeutic rationale.	Negative phase II clinical evidence. This row is important because the reviewer specifically requested unsuccessful studies.	[46]
Pembrolizumab with or without entinostat	HDAC1/3 + PD-1	Phase II window-of-opportunity study, NCT03978624	Cisplatin-ineligible cT2-T4aN0M0 muscle-invasive urothelial cancer before radical cystectomy or trimodality therapy	Immune gene signature, neoantigen expression, TCR clonality and tumor immune profiling	Twenty patients were treated. Among patients undergoing radical cystectomy, pathologic response rate was 43% and pathologic complete response was 29% in both treatment arms, providing no clear clinical-response advantage from adding entinostat in this small dataset.	Grade 3 or greater treatment-related adverse events occurred in 10%; no definitive-treatment delay was attributed to study treatment. Primary translational analyses were still ongoing in the 2025 report.	Preliminary phase II evidence. Combination is feasible, but incremental clinical benefit of entinostat is unproven.	[23,49]
JQ1, alone or with class I HDAC inhibition	BET/BRD4, with or without HDAC	Preclinical	Human urothelial carcinoma cell lines	No validated patient-selection biomarker	BET inhibition suppressed oncogenic transcription and growth experimentally; combined JQ1 and romidepsin produced synergistic apoptosis across urothelial carcinoma cell lines.	Cell-line evidence does not establish efficacy, safety or patient selection in UBC.	Preclinical only. Do not describe BET inhibition as clinically “promising” or actionable.	[26,27]

Collectively, the therapeutic evidence indicates a substantial gap between epigenetic dependency identified experimentally and demonstrated clinical benefit in UBC. KDM6A/EZH2 biology provides a comparatively strong mechanistic example, but clinical predictive validation remains incomplete. HDAC- and DNMT-directed studies have produced mixed or negative findings, including meaningful toxicity, while BET-directed treatment remains largely preclinical. The most defensible current role of these strategies is therefore as investigational approaches requiring molecularly informed clinical evaluation rather than as established components of UBC treatment.

## Emerging technologies

Emerging epigenomic technologies may help address several limitations that currently impede mechanistic and clinical translation in UBC. Bulk genomic and methylation studies identify recurrent alterations at the population level but may obscure intratumoral cell-state heterogeneity and the molecular context in which individual epigenetic dependencies arise. Single-cell, spatial, and longitudinal approaches can provide greater resolution of chromatin accessibility, regulatory states, tumor immune interactions, and treatment-associated adaptation. Their value, however, should be evaluated according to whether they generate reproducible biological insight or improve biomarker validation, patient stratification, treatment selection, or disease monitoring rather than being assumed to provide clinical actionability by virtue of technological resolution alone.

Single-cell epigenomic technologies, including scATAC-seq and multimodal single-cell approaches, allow characterization of chromatin accessibility in individual cells [50,51]. This is particularly valuable in UBC, where intratumoral heterogeneity is a defining feature and treatment-resistant subpopulations may be masked in bulk datasets. By resolving rare or treatment-associated cellular states, single-cell approaches may refine biological classification and generate candidate mechanisms of invasion, immune exclusion, or therapeutic resistance. Such observations require functional and clinical validation before they can be regarded as therapeutic vulnerabilities [52].

Spatial epigenomic and imaging-based methods extend these insights by preserving tissue architecture. This allows researchers to map regulatory states within their anatomical context and examine interactions between tumor cells, stromal populations, and immune infiltrates. In bladder cancer, where tumor–microenvironment relationships strongly influence progression and treatment response, this spatial dimension is especially important [53].

At the same time, liquid biopsy approaches are increasingly relevant because UBC is particularly well suited to urine-based biomarker development. Detection of tumor-derived methylated DNA, chromatin-associated signals, or other circulating molecular features in urine or plasma offers opportunities for early diagnosis, recurrence monitoring, and treatment surveillance in a minimally invasive manner. However, urine- and plasma-based epigenetic biomarkers should be considered complementary to cystoscopy, cytology, imaging, and tissue-based molecular profiling rather than universal replacements for established diagnostic or surveillance pathways. Finally, AI-driven multi-omics integration is becoming critical for synthesizing the complexity of epigenetic data. Integrating chromatin accessibility, methylation, transcriptomic, proteomic, and clinical information can help identify robust biomarker signatures, classify biologically meaningful subtypes, and predict therapeutic response more accurately than single-layer analyses. Advanced epigenomic technologies may improve the resolution of tumor-state mapping, microenvironmental analysis, biomarker discovery, and patient stratification. Their clinical value will depend on reproducible validation, integration with existing diagnostic pathways, and demonstration that multi-omics information can improve treatment selection or longitudinal monitoring (Figure 3).

This conceptual figure presents an authors’ proposed framework integrating tissue, urine, and plasma sampling with sequencing, single-cell, spatial, imaging, and computational approaches for UBC epigenetic research. The individual technologies and translational applications shown are supported by different bodies of evidence and should not be interpreted as components of a single clinically validated workflow.

## Clinical translation

A major challenge in urothelial bladder cancer research is the translation of epigenetic discovery into clinically meaningful tools and treatment strategies [12]. The most immediate opportunities lie in three areas: biomarker development, molecular stratification, and therapeutic combination design.

The clinical translation of UBC epigenetics should be evaluated according to the level of evidence supporting each application. A recurrent molecular alteration may establish biological relevance without establishing biomarker validity, while analytical detection of an epigenetic signature does not by itself demonstrate clinical utility. Similarly, preclinical sensitivity to an epigenetic inhibitor should not be equated with clinical actionability. Translation therefore requires sequential evidence of biological validity, analytical reproducibility, prospective clinical validation, and demonstration that biomarker-informed management improves diagnosis, surveillance, treatment selection, or patient outcome.

Among epigenetic biomarkers, urine DNA-methylation assays currently have the strongest UBC-specific clinical evidence, including prospective studies evaluating detection and surveillance applications [31–33]. Their potential clinical role remains assay- and use-case-specific, and performance should be compared directly with established diagnostic and surveillance pathways. Plasma cfDNA methylation remains less mature and is currently supported primarily by exploratory translational evidence [35].

Epigenetic alterations may also contribute to molecular stratification, but recurrent mutation, enhancer state, or immune-associated chromatin phenotype should not be assumed to predict treatment benefit [54]. Clinical utility requires prospective demonstration that an epigenetic biomarker identifies a reproducible patient subgroup and improves treatment selection or patient outcomes beyond established clinicopathologic and genomic variables. Clinical translation increasingly depends on combination therapy design. Because epigenetic drugs can alter tumor cell state, transcriptional accessibility, and immunogenicity, their greatest value may be in reprogramming tumors to become more responsive to other interventions [55,56]. This is particularly important for patients with resistant or immune-refractory disease. In this framework, epigenetic modulation is not merely cytotoxic treatment; it is a strategy for reshaping the biological context in which other therapies act [57,58].

## Future perspectives

Future progress in urothelial bladder cancer epigenetics will depend on moving from broad descriptive associations toward precise identification of driver epigenetic events, state-dependent vulnerabilities, and clinically useful predictive markers. A central challenge is that epigenetic states are dynamic, context-dependent, and strongly influenced by treatment exposure and microenvironmental conditions. As a result, the same tumor may occupy multiple regulatory states over time, complicating both classification and therapeutic intervention.

One priority is the development of more refined molecular frameworks that integrate chromatin remodeling defects, histone modification patterns, DNA methylation states, enhancer topology, transcriptomic outputs, and immune contexture. Such integrated models could help define biologically and clinically meaningful subtypes that are more informative than genomic classification alone.

A second priority is improved therapeutic precision. Current epigenetic agents often affect broad chromatin programs, which may limit selectivity and contribute to off-target toxicity. Future strategies will require better identification of tumors that are genuinely dependent on specific chromatin regulators or enhancer networks. This may be particularly important for exploiting synthetic lethality, where therapeutic efficacy depends on precise molecular context.

A third challenge is the need for longitudinal monitoring. Because chromatin states evolve during therapy, single time-point profiling is unlikely to capture the full biology of resistance. Repeated assessment through tissue and liquid biopsy, combined with single-cell and spatial analyses where feasible, may provide more accurate insight into adaptive reprogramming and resistance emergence. Ultimately, the field must address a central conceptual question: which epigenetic changes are true drivers of malignant behavior, and which are secondary or passenger consequences of transformation? Resolving this will require stronger functional validation, improved experimental models, and carefully integrated translational studies. The future of the field lies not only in cataloguing epigenetic abnormalities, but in converting them into actionable biological principles that improve patient care.

### Limitations

This review has several limitations. First, it is a structured narrative review rather than a systematic review or meta-analysis, and study inclusion therefore involves interpretive selection. Second, the evidence base is heterogeneous with respect to disease stage, molecular subtype, specimen type, experimental model, epigenetic assay, and therapeutic setting, limiting direct comparison among studies. Third, mechanistic evidence for some epigenetic regulators remains derived partly from non-UBC systems and should not be assumed to apply uniformly to urothelial tumors. Fourth, many biomarker studies are retrospective or lack independent prospective validation, while clinical studies of epigenetic therapies are generally small and early phase. Publication bias toward positive mechanistic or biomarker findings is also possible. Finally, rapid advances in single-cell, spatial, liquid-biopsy, and epigenomic technologies mean that the translational evidence base continues to evolve. These limitations reinforce the need to distinguish established UBC-specific evidence from extrapolation and hypothesis-generating observations throughout this review.

## Conclusion

Epigenetic dysregulation is an important component of urothelial bladder cancer biology, but the strength of evidence differs markedly among molecular mechanisms, biomarkers, and therapeutic targets. Functional studies support causal roles for selected chromatin regulators, including KDM6A and KMT2C/KMT2D, whereas many other reported epigenetic alterations remain primarily associative. DNA-methylation biomarkers, particularly those measured in urine, represent one of the most advanced translational applications, although prospective validation and assay standardization remain necessary. Plasma cfDNA methylation offers complementary potential but remains less clinically mature. Therapeutically, preclinical dependencies involving EZH2, HDAC, BET, and DNMT pathways have not yet translated into routine epigenetically selected treatment for UBC, and negative clinical findings emphasize the need for cautious interpretation. Progress will depend on functional validation, biomarker-driven clinical trials, longitudinal sampling, and integration of tissue and liquid-biopsy epigenomics to determine which alterations are biologically consequential and clinically useful.

## Data Availability

Data sharing is not applicable to this article because no new datasets were generated or analysed.

## Abbreviations

UBC: Urothelial Bladder Cancer
NMIBC: Non-Muscle-Invasive Bladder Cancer
MIBC: Muscle-Invasive Bladder Cancer
SWI/SNF: SWItch/Sucrose Non-Fermentable Chromatin Remodeling Complex
DNMT: DNA Methyltransferase
HDAC: Histone Deacetylase
EZH2: Enhancer of Zeste Homolog 2
KMT2C/D: Lysine Methyltransferase 2C/D
KDM6A: Lysine Demethylase 6A
EP300: E1A Binding Protein p300
CREBBP: CREB Binding Protein
BET: Bromodomain and Extra-Terminal Domain Proteins
ATAC-seq: Assay for Transposase-Accessible Chromatin using Sequencing
ChIP-seq: Chromatin Immunoprecipitation Sequencing
scRNA-seq: Single-cell RNA Sequencing
scATAC-seq: Single-cell ATAC Sequencing
MHC: Major Histocompatibility Complex), PD-1 (programmed cell death protein 1
AI: Artificial Intelligence
PRC2: Polycomb repressive complex 2
NK: natural killer
BCG: Bacillus Calmette-Guérin
cfDNA: cell-free DNA
PPV: positive predictive value, NPV, negative predictive value
AUC: area under the curve
RECIST: Response Evaluation Criteria in Solid Tumors
ORR: objective response rate
PFS: progression-free survival
G-CSF: granulocyte colony-stimulating factor
UC: urothelial carcinoma
RCC: renal cell carcinoma
TCR: T-cell receptor
